# Investigation of vaginal microbiota in sexually active women using hormonal contraceptives in Pakistan

**DOI:** 10.1186/1471-2490-12-22

**Published:** 2012-08-18

**Authors:** Yasmeen Faiz Kazi, Sobia Saleem, Nasreen Kazi

**Affiliations:** 1 Department of Microbiology, Shah Abdul Latif University, Khairpur, Sindh, Pakistan; 2 Department of Pharmacology, Liaqat University of Medical and Health Sciences, Jamshoro, Sindh, Pakistan

## Abstract

**Background:**

Previous studies report association of contraceptives with moderate increase in urinary tract infection among sexually active premenopausal women. The aim of our study was to find out whether the use of hormonal contraceptives has any effect on microbiota of the vagina in the contraceptives users in Khairpur Sindh Pakistan.

**Methods:**

A prospective study in woman population of Khairpur Sindh Pakistan aged 20–30 years and 31–40 years, using Hormonal contraceptives was carried out. High vaginal swab samples (n = 100) were collected from the test populations as well as control group (n = 100) and investigated for vaginal microbial flora using standard microbiological and biochemical techniques.

**Results:**

Vaginal swabs culturing from hormonal contraceptives users in the age group 20–30 years showed statistically insignificant *Candida sp (*10% samples), and statistically significant (*p* < 0.05) *Staphylococcus saprophyticus.* (18% samples), *Streptococcus agalactiae* (23% samples), *Escherichia coli* (28% samples) and *Lactobacillus fermentum* (32% samples). In the age group 31–40 years, statistically significant percentage of samples (*p* < 0.05) showed *Lactobacillus fermentum* (28%), *Candida* sp (24%)*,* and *E. coli,* (24%) where statistically insignificant samples showed *Staphylococcus saprophyticus* (13%) and *Streptococcus agalactiae* (11%).

**Conclusions:**

The use of hormonal contraceptives alters the normal microbiota of vagina in women according to the age. *Lactobacillus fermentum* appeared as the predominant species followed by *E. coli* among the age group of 20–30 years and, *Lactobacillus fermentum*, *Candida sp* and *E. coli* as predominant among women of age group 31–40 years when compared to corresponding control groups. An inverse relationship between *E. coli* and *Lactobacillus fermentum* was observed in the women aged 20–30 years.

## Background

Hormonal contraception (HC) mentions to birth control methods that act on the endocrine system.

Previous studies identified urinary tract infection (UTI) as a complication of contraceptive use [1,2]. Around 17 million women per year in the United States suffer UTI and costs billions to the economy [3]. Methods of contraception have suffered a set back in improving the health of women because of concerns in many countries about the mechanism and side effects of the contraceptives [4]. As the population is growing most rapidly in the developing countries including Pakistan, it has put much emphasis to its ‘population planning prograrmme and despite spending a significant budget, the objectives are yet not achieved [5]. One of the reasons is fear of UTI, a serious health problem affecting millions of women each year. UTI are the second most common type of infection. Women are especially prone to UTI and one woman in five develops a UTI during her lifetime [1]. UTI as a side effect of HC usage has not been previously documented in Khairpur. This study is aimed at addressing this gap in knowledge by analyzing effect of hormonal contraceptives on vaginal **microbiota** of women of reproductive age using HC in Khairpur city.

## Methods

The samples were randomly collected from non-pregnant pre- menopausal women aged 20–30 and 31–40 years (n = 100 for each group) using hormonal **contraceptions** and from control group (women not using contraceptives; n = 100 for each group). The samples were taken from different hospitals of Khairpur city, Sindh Pakistan. The hospital include: Civil Hospital, Lady Wellington Hospital, Reproductive Health Services and Maternity Home Luqman. An informed consent was taken prior to the sample collection. This study was approved by the District health committee Khairpur.

### High vaginal swab collection

Vaginal swabs specimens from contraceptive users and control group were collected using commercial sterile cotton swabs by inserting approximately 1 inch into vagina and immediately transported to the laboratory in Amies transport medium (Chem Malaysia) by keeping them in ice box. Microbiological investigations were carried out in the Diagnostic and research center department of Microbiology Shah Abdul Latif University Khairpur. The swabs were aseptically streaked on various culture media such as Blood agar (Oxoid) **containing 10% (v/v) defibrinated sheep blood,** Chocolate agar (Oxoid), Mac Conkey’s agar (Oxoid), Nutrient agar (Oxoid) and Saboureaud dextrose agar (Oxoid) and incubated aerobically at 37°C for 24 h. After incubation, results were recorded semi-quantitatively as described by Gupta et al. [6] as 0 (no growth), 1+ (colonies on first streak zone), 2+ (colonies on first and second streak zone, 3+ (colonies on first, second and third streak zone) and 4+ (colonies on first, second, third and fourth streak zone). Colony morphology of isolates was inspected and recorded. Gram stain and biochemical tests were performed using standard microbiological techniques. Pure cultures of the isolated microorganisms were preserved in sterile glycerol broth (16% v/v in nutrient broth) for identification by standard biochemical tests. *E*. *coli ***was initially isolated on Mac Conkey’s agar and then in nutrient agar and** identified using API-20 E (bioMerieux France) **according to manufacturer’s instructions**. *Staphylococcus* sp. **was grown in nutrient agar** and identified on the bases of catalase test, coagulase test, hemolysis on blood agar **containing 10% (v/v) defibrinated sheep blood** and novobiocin (Hugo diagnostics; 5 micro gram disc) sensitivity test using disc diffusion method; *Streptococcus* sp. was **grown in nutrient agar** and identified on the bases of catalase test, hemolysis on sheep blood agar **containing 10% (v/v) defibrinated sheep blood,** bile solubility test and bacitracin (Oxoid) sensitivity test. *Lactobacillus* sp. **was grown in nutrient agar (pH 5.5) in the presence of 5% CO**_**2**_**and then** identified on the bases of catalase test, and glucose fermenting activity [7-10].

### Germ tube test

Oval budding cells were investigated for identity as *Candida albicans* by germ tube test [9]. A 500 μl of human serum was placed in to a small test tube to observe tube like outgrowths from the cells (known as germ tube), indicative of *C. albicans*. Using a sterile wire loop, the serum was inoculated with a yeast colony from SDA plate. The tube was placed in a water bath at 37°C for 3 h. The wet mount preparation was examined using the 10X and 40X objectives with the condenser iris diaphragm closed sufficiently to give good contrast.

## Statistical analysis

The data were analyzed using the Statistical Package for Social Sciences (SPSS) version 10 and the Microsoft Excel (MS) software program. The proportions of microorganisms with age group of HC user and control group were calculated as a percentage. The degree of association of each type of microorganism from HC user of each age group with use of hormonal contraception were determined using Mc Nemar’s test. Tables were constructed to present the results. Statistical significance was set at the 95% confidence level CI, confidence interval) or at a *p*-value of less than or equal to 0.05 (*p* value ≤ 0.05).

## Results

Vaginal swabs specimen of contraceptive users from different hospitals in Khairpur city were investigated in both age groups and control group. Gram staining showed G + oval budding cells, G + cocci in bunches, G + cocci in chains*,* G- coccobacilli, and G + filamentous rods. The isolated microorganisms were identified by biochemical tests (Table 1). Further identification of *E. coli* was performed by API-20E. *Staphylococcus* sp. was further characterized on the bases of coagulase test, hemolysis on blood agar and novobiocin sensitivity. Confirming of the specie was carried out using novobiocin sensitivity test. The *Staphylococcus* sp. was coagulae- negative; showed beta hemolysis on blood agar and was resistant to novobiocin (zone size < 17 mm) therefore it was identified as *Staphylococcus saprophyticus.* The *Streptococcus* sp. showed catalase negative reaction, beta hemolysis on blood agar and was bile insoluble. Bacitracin resistance response indicated that this organism was *Streptococcus agalactiae* which belong to group B. The *Lactobacillus* sp. showed catalase negative reaction and fermented glucose with acid and gas which characterized this organism as *Lactobaccilus fermentum*. In case of *Candida* sp. further characterization was not performed as the interest was to isolate *C. albicans* which was ruled out by negative germ tube test. Semi-quantitative grading and isolation percentage was calculated for test and control groups. Tables 2 and 3 show the semi quantitative grading and isolation percentages from women aged 20–30 and 31–40 years using HC and from control group respectively. The data showed statistically significant difference in the trend of isolation and semi- quantitative grading of vaginal bacteria among the age group 20–30 years when compared to the control group. *Staphylococcus saprophyticus.* was found in 18% of women using HC and 9% of the controls (*p* < 0.05), *Streptococcus agalactiae* in 23% and 10% of control (*p* < 0.05), *E. coli* in 28% and 6% of control (*p* < 0.05), *Lactobacillus fermentum* in 32% women and 12% of control (*p* < 0.05). Only the isolation percentages of *Candida* sp from HC users and control shown statistically insignificant difference (*p* <0.05) and the trend remained similar.

**Table 1:** Initial biochemical characterization of the vaginal swab isolates from women using hormonal contraceptives and control group

**Strain**	**Isolated microorganism**	**Germ tube test**	**Biochemical tests**			**Identified microorganism**
			***Catalase***	***Oxidase***	***Coagulase***	
1	G + oval budding cells	Negative	N/A	N/A	N/A	*Candida* sp.
2	G + cocci in bunches	N/A	+	−	−	*Staphylococcus saprophyticus.*
3	G + cocci in chains	N/A	−	−	−	*Streptococcus agalactiae*.
4	G- coccobacilli	N/A	+	−	−	*E. coli*
5	G- filamentous rods	N/A	−	−	−	*Lactobacillus fermentum*

Vaginal swabs were obtained from women using hormonal contraceptives and processed. The table shows the isolated and identified microorganisms from the age groups: 20–30 years and 31–40 years (n = 100 for each group). N/A denotes ‘not applicable’.

**Table 2:** Determination of the percentage of microorganisms isolated from vaginal swabs of women using hormonal contraceptives

**Strain**	**Cultural characters**	**Identified Microorganisms**	**Total percentage (%)in various age groups**	
			**20–30 years**	**31–40 years**
**1**	Oval budding cells	*Candida* sp*.*	10 (1 +)	24 (3+)
**2**	G + cocci	*Staphylococcus* *saprophyticus*.	18 (2 +)	13 (1+)
**3**	G + cocci in chains	*Streptococcus agalactiae*	23 (3 +)	11 (1 +)
**4**	G- coccobacilli	*E. coli*	28 (4+)	24 (3 +)
**5**	G + long filamentous rods	*Lactobacillus fermentum*	32 (2 +)	28 (4 +)

Vaginal swabs were obtained from women using hormonal contraceptives and processed. The table shows the percentages and semi-quantitative grading in parenthesis of isolated and identified microorganisms from age groups: 20–30 years and 31–40 years and control groups(n = 100 for each group).

**Table 3:** Determination of the percentage of microorganisms from vaginal swabs of control group

**Strain**	**Cultural characters**	**Identified Microorganisms**	**Total percentage (%)in various age groups**	
			**20–30**	**31–40**
**1**	Oval budding cells	*Candida* sp.	13 (1 +)	10 (1 +)
**2**	G + cocci	*Staphylococcus saprophyticus.*	09 (1 +)	12 (1+)
**3**	G + cocci in chains	*Streptococcus agalactiae*	10 (1+)	08 (1 +)
**4**	G- coccobacilli	*E. coli*	06 (1+)	04 (1 +)
**5**	G+,long filamentous rods	*Lactobacillus fermentum* .	12 (1 +)	16 (1 +)

Vaginal swabs were obtained from women of control group and processed. The table shows the percentages and semi-quantitative grading in parenthesis of isolated and identified microorganism from 20–30 years and 31–40 years of age (n = 100 for each group).

**Figure 1:**
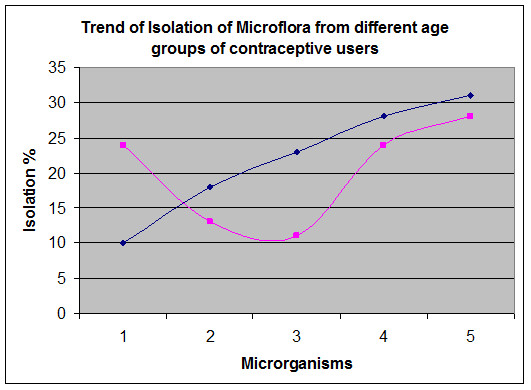
**Vaginal swabs were obtained from women using hormonal contraceptives and processed.** The graph show the trend of isolated and identified each microorganism isolated. Black line shows microorganisms from 20–30 years, pink line shows microorganisms from 31–40 years of age. 1*. Candida* sp, 2. *Staphylococcus saprophyticus,* 3*. Streptococcus agalactiae,* 4. *Escherichia coli,* 5. *Lactobacillus fermentum.*

**Figure 2:**
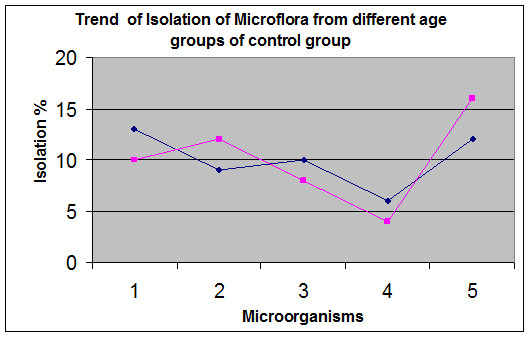
**Vaginal swabs were obtained from control group not using hormonal contraceptives were processed.** The graph shows the trend of isolated and identified microorganism. Black line shows microorganism from 20–30 years, pink line shows microorganisms from 31–40 years of age. 1. *Candida* sp, 2*.Staphylococcus saprophyticus,* 3. *Streptococcus agalactiae,* 4. *Escherichia. coli,* 5. *Lactobacillus fermentum.*

In the women of age group 31–40 years, the trend of isolation showed statistically significant difference (*p* <0.05) among HC users and respective control group. Here the *Candida* sp was present in 24% women and 10% of control (*p* < 0.05), *E. coli* in 24% women and 4% of control (*p* < 0.05) and *Lactobacillus fermentum* in 28% women and 16% of control (*p* < 0.05). Statistically insignificant difference was found between HC users and control for the isolated *Staphylococcus* and *Streptococcus* species. Trends of isolation rate (%) revealed that *Lactobacillus fermentum* was the predominant vaginal flora in women of age group 20–30 years, where the *Candida* sp was predominant in women of age group 31–40 years as shown in Figure 1. The control group showed similar trend in both age groups (20–30, 31–40 years) as shown in Figure 2.

## Discussion

This study was undertaken to investigate the effect of oral contraceptives on vaginal ecology of the users of two age groups. Five microbial strains were isolated and evaluated as reference index strains. So far, there is no known prospective study that has ascertained the role of contraceptives in women of different age groups using hormonal contraceptives in Khairpur Sindh Pakistan.

In present study, we report that the overall percentage of vaginal microbiota amongst the contraceptive users of age group 20–30 and 31 to 40 years was high (45%) and this finding is consistent with other researchers [6,11-13]. **The reason why the study was confined to the microbial species reported here is that these microorganisms represent opportunistic group (except *Lactobacillus fermentum ***) **and etiology of most urinary tract infections have been attributed to these microbiota.**

Our study revealed that there was insignificant difference in type and trend of microorganisms among both control groups. Whereas, a significant difference was observed in the trend of microorganisms particularly for *Candida* sp and *Lactobacillus fermentum* between age groups 20–30 and 31–40 years that indicates the role of age factor in vaginal ecology. **Though *Lactobacillus fermentum *was isolated from both age groups, the preventive role remained uncertain in the presence of other species isolated in our study. *Lactobacillus fermentum *probiotic strains have been used with poor results in urogenital infection**[14]**, and this may have been the cause of overgrowth of the other microorganisms. However, racial variation in terms of vaginal *Lactobacillus *species due to geographical region are reported**[15]**.**

There was no significant difference in isolation percentage of *Candida* sp from test and control group of women aged 20–30 years which is consistent with the findings of Peddie, et al., [16] who reported no significant difference in contraceptives user and non-users when *C. albicans* was investigated. On the other hand, statistically significant difference in *Candida* sp compared to control was observed in women of age group 31–40 years which is not in agreement with the same study [16]. This elaborates the importance of age factor as a crucial variable and determinant for vaginal ecology. An inverse relationship was observed between *E. coli* and *Lactobacillus fermentum*. in women aged 20–30 years which is consistent with the findings of Gupta, et al. [6] however, no such association was established in our study for the group of women aged 31–40 year. The limitations of our study include unavailability of culture-independent isolation of the vaginal microbiota. **Although other species of *Lactobacilli *have been used as probiotic against UTI**[17]**, the higher number of vaginal microbial flora in presence of *Lactobacilli fermentum *amongst women using HC indicate diminished role of this species and this may pose increased risk of the development of UTI in contraceptive users compared to non-users particularly in women above 30 years of age.** Among the premenopausal women aged 37 to 54 years, with a history of oral contraception, a modest increase in the UTI has recently been published [1].

It has been reported that beside the suppression of ovulation, other peripheral manifestations in contraceptive users are thickening of cervical mucus, change in muscle tone and cervical endometrium [11,18]. Although these parameters were not investigated in the present study, the findings indicate that these factors may have been the cause of colonization of vagina with altered, higher percentages of vaginal microbiota found in our study and that these effects may become more enhanced in the age above 30 years.

## **Conclusion**

On the basis of the findings of this study it can be concluded that the age of a women using hormonal contraceptives is an important factor for the alteration in vaginal microbiota. The present study provides important information regarding the vaginal ecology of women using hormonal contraceptives particularly after 30 years of age. A greater attention to be paid to the family planning methods. The women using hormonal contraceptives may regularly be monitored for alterations in vaginal ecology in order to keep the UTI minimized.

## Competing interest

The authors declare that they have no competing interests.

## Author’s contributions

YFK conceived and coordinated the study, SS processed the samples, NK collected the HVS samples. All authors read and approved the final manuscript.

## Pre-publication history

The pre-publication history for this paper can be accessed here:

http://www.biomedcentral.com/1471-2490/12/22/prepub
